# Draft Genome and Complete *Hox*-Cluster Characterization of the Sterlet (*Acipenser ruthenus*)

**DOI:** 10.3389/fgene.2019.00776

**Published:** 2019-09-05

**Authors:** Peilin Cheng, Yu Huang, Hao Du, Chuangju Li, Yunyun Lv, Rui Ruan, Huan Ye, Chao Bian, Xinxin You, Junmin Xu, Xufang Liang, Qiong Shi, Qiwei Wei

**Affiliations:** ^1^Key Laboratory of Freshwater Biodiversity Conservation, Ministry of Agriculture of China, Yangtze River Fisheries Research Institute, Chinese Academy of Fishery Sciences, Wuhan, China; ^2^College of Fisheries, Chinese Perch Research Center, Huazhong Agricultural University, Wuhan, China; ^3^Shenzhen Key Lab of Marine Genomics, Guangdong Provincial Key Lab of Molecular Breeding in Marine Economic Animals, Academy of Marine Sciences, BGI Marine, Shenzhen, China; ^4^BGI Education Center, University of Chinese Academy of Sciences, Shenzhen, China; ^5^School of Veterinary Medicine, Rakuno Gakuen University, Ebetsu, Japan

**Keywords:** sterlet, sturgeon, genome, *hox*, lineage-specific whole genome duplication

## Abstract

**Background:** Sturgeons (Chondrostei: Acipenseridae) are a group of “living fossil” fishes at a basal position among Actinopteri. They have raised great public interest due to their special evolutionary position, species conservation challenges, as well as their highly-prized eggs (caviar). The sterlet, *Acipenser ruthenus*, is a relatively small-sized member of sturgeons and has been widely distributing in both Europe and Asia. In this study, we performed whole genome sequencing, *de novo* assembly and gene annotation of the tarlet to construct its draft genome.

**Findings:** We finally obtained a 1.83-Gb genome assembly (BUSCO completeness of 81.6%) from a total of 316.8-Gb raw reads generated by an Illumina Hiseq 2500 platform. The scaffold N50 and contig N50 values reached 191.06 and 18.88 kb, respectively. The sterlet genome was predicted to be comprised of 42.84% repeated sequences and to contain 22,184 protein-coding genes, of which 21,112 (95.17%) have been functionally annotated with at least one hit in public databases. A genetic phylogeny demonstrated that the sterlet is situated in the basal position among ray-finned fishes and 4dTv analysis estimated that a recent whole genome duplication occurred 21.3 million years ago. Moreover, seven *Hox* clusters carrying 68 *Hox* genes were characterized in the sterlet. Phylogeny of *Hox*A clusters in the sterlet and American paddlefish divided these sturgeons into two groups, confirming the independence of each lineage-specific genome duplication in Acipenseridae and Polyodontidae.

**Conclusions:** This draft genome makes up for the lack of genomic and molecular data of the sterlet and its *Hox* clusters. It also provides a genetic basis for further investigation of lineage-specific genome duplication and the early evolution of ray-finned fishes.

## Introduction

Sturgeons (Acipenseridae, Acipenseriformes) have long been considered as an interesting group of fishes due to their commercial value and conservational challenges (Wei et al., 2011). They have also drawn noteworthy attention due to occupying a basal position on the phylogenetic tree of ray-finned fishes. It is estimated that the origin of sturgeons dates back to approximately 350 million years ago (Mya), which is even earlier than the origins of Holostei (bowfin and gars) and Teleostei (teleosts) (Hughes et al., 2018). Therefore, sturgeons did not the teleost-specific genome duplication (TGD) event that happened around 320 Mya (Jaillon et al., 2004). However, there are clear evidences based on molecular markers, chromosome numbers and inferred ploidy levels that they have experienced their own lineage-specific polyploidizations with one or more rounds of genome duplication (GD; Crow et al., 2012), resulting in complex genome structures and the widest range of chromosome numbers among all vertebrates (Havelka et al., 2016). However, little is known about Acipenseridae-specific GD and its consequences due to a lack of sturgeon genome sequences.

This special whole genome duplication (WGD) event has also provided new genetic material to generate phenotypic diversity among sturgeons. However, sturgeons have quite limited species diversity with exceedingly fast overall rates of body size evolution, serving as an interesting exception to the phenotypic ‘evolvability’ hypothesis (Rabosky et al., 2013). As one of the earliest evolved fish groups among ray-finned fishes, sturgeons still retain many shark-like features such as a cartilaginous skeleton and heterocercal tail, and the extant species look conspicuously similar to their fossil counterparts, suggesting that there has been of body-shape evolution (Rabosky et al., 2013). Therefore, sturgeons represent an ideal evolutionary group to investigate the complicated relationship between phenotypes and the polyploidy genomes caused by WGD. Meanwhile, *Hox* genes, encoding a distinct class of transcription factors associated with axial patterning and appendages development, have been often among the first list for examination to understand their roles in evolution of vertebrate body plans and novelty (Amemiya et al., 2010; Crow et al., 2012).

The sterlet (*Acipenser ruthenus*, Linnaeus, 1758) is a famous representative of sturgeon species, well-known for its relatively small body size and wide distribution in comparison to other sturgeons. Composed of 120 chromosomes, the sterlet genome has both diploid and tetraploid chromosome segments (Romanenko et al., 2015); however, various chromosomes are unequally involved in the multiple interchromosomal rearrangements after the GD event (Andreyushkova et al., 2017). In this study, we performed whole genome sequencing of the sterlet and generated a draft genome assembly of a sturgeon for the first time. We also constructed a fossil-calibrated phylogenetic tree, estimated the occurrence time of the sturgeon-specific GD (although it is unclear how many members in this family have experienced such an independent lineage-specific GD, considering that this is the first sturgeon with public genome sequences) and retrieved the complete *Hox* clusters to preliminarily reveal the early evolutionary history of ray-finned fishes.

### Value of the Data

- This is the first genome report of a sturgeon. The sterlet genome was determined to be in size with a scaffold N50 of 191.06 kb. Our draft assembly contains 784 Mb (42.84% of the genome) of repeats and 22,184 protein-coding genes.
- The time-calibrated phylogenetic tree showed a most basal position of sterlet in Actinopterygii (ray-finned fishes) and dated the origin of the sterlet back to 358 Mya, which is extremely close to the Late Devonian Extinction that happened approximately 358,9 Mya.
- 4dTv analysis showed that the sturgeon-specific GD event happened about 21.3 Mya, close to the estimated occurrence time (42 Mya) of paddlefish-specific GD event, regardless of the independence of these two WGD events.
- Seven *Hox* clusters including 68 *Hox* genes were identified in the sterlet genome. Phylogeny of *Hox*A clusters of the sterlet and American paddlefish divided these sturgeons into two groups, suggesting that the WGD events happened independently in these two sturgeon species.

## Materials and Methods

### Sample Processing

The sequenced sterlet (an immature juvenile, about 2.5 years old, 56.8 cm in length, weighing 0.8 kg) was artificially cultured at Taihu Station, Yangtze River Fisheries Research Institute, Chinese Academy of Fisheries Sciences, China. First, we obtained 10 mL of blood from the caudal vertebral vessels (without sacrificing the fish), but the sample was only sufficient for transcriptome sequencing. Subsequently, we had to anesthetize and sacrifice the fish to collected 30 g of skeletal muscle in order to obtain enough DNA for genome sequencing. All vouchers were deposited in China National GeneBank with accession numbers of WH20161125002-MU (muscle) and -BL (blood). All experiments were carried out in accordance with the guidelines of the Animal Ethics Committee of Yangtze River Fisheries Research Institute of Chinese Academy of Fishery Sciences (No. YFI-01).

### Genome Sequencing and Assembly

We applied whole-genome shotgun sequencing to generate short paired-end reads (125 or 150 bp) by constructing a series of short-insert (270, 500, and 800 bp) or long-insert (2, 5, 10, and 20 kb) libraries (**Supplementary Figure 1**) and sequencing on a Hiseq 2500 platform (Illumina, San Diego, CA, USA). Raw reads were subsequently pre-processed by SOAPfilter software (Luo et al., 2012) to trim five bases at the 5’ end of all reads and to discard the low-quality reads (quality value <20) and those reads with many nonsequenced bases (N > 10). Subsequently, the 17-mer depth frequency distribution method was employed to estimate the genome size of the sterlet using data from short-insert libraries according to the following formula: genome size = total number of k-mers/peak value of k-mer frequency distribution (Li et al., 2010). Clean reads from all the seven libraries were assembled into contigs and scaffolds using SOAPdenovo v2.04 (Luo et al., 2012) with optimized parameters (pregraph -K 41 -d 1; contig –M 3; scaff -F; others as the default). Finally, gaps in the scaffolds were successively filled by using Kgf and GapCloser (Luo et al., 2012) with clean reads from short-insert libraries. Completeness of the final genome assembly and the entire gene set was assessed by BUSCO (Simão et al., 2015).

### Repeat-Sequence Prediction and Gene Annotation

A *de novo* repeat library for the sterlet was constructed by a combination of RepeatModeler v1.05 (RepeatModeler, RRID: SCR_015027) and LTR_FINDER v1.0.6 (Xu and Wang, 2007). Known and *de novo* transposable elements (TEs) in the assembled genome were identified by RepeatMasker v4.0.6 (RepeatMasker, RRID : SCR_012954) using both the RepBase v21.01 (Jurka et al., 2005) and the *de novo* repeat library. RepeatProteinMask v3.3.0 (Chen, 2004) was then used to identify the TE relevant proteins. Meanwhile, tandem repeats were predicted by using Tandem Repeats Finder (TRF) v4.07b (Benson, 1999), and Tandem Repeats Analysis Program (Sobreira et al., 2006) was used to select candidate microsatellite markers from the TRF output.

Gene models in the sterlet genome were predicted by an integrated strategy of three methods. For homology annotation, we downloaded published protein sequences of ten representative vertebrates including zebrafish (*Danio rerio*), spotted gar (*Lepisosteus oculatus*), elephant shark (*Callorhinchus milii*), sea lamprey (*Petromyzon marinus*), medaka (*Oryzias latipes*), Nile tilapia (*Oreochromis niloticus*), three-spined stickleback (*Gasterosteus aculeatus*), Atlantic cod (*Gadus morhua*), fugu (*Takifugu rubripes*) and spotted green pufferfish (*Tetraodon nigroviridis*), and aligned them against the assembly of the sterlet genome using BLAST (Altschul et al., 1990) with tblastn mode and an e-value of 1e-5. SOLAR (Yu et al., 2006) was subsequently employed to select the best hit for each alignment. For *ab initio* prediction, the sterlet genome assembly was masked according to the previously identified repeated sequences and was then scanned using AUGUSTUS v3.2.3 (Stanke et al., 2006) and GENSCAN v1.0 (Burge and Karlin, 1997) to predict gene structures. For transcriptome-based annotation, we sequenced a blood transcriptome on a Hiseq X10 platform (Illumina), mapped the reads to the genome scaffolds using TopHat v2.0.13 (Trapnell et al., 2009) and assembled them into transcripts using Cufflinks v2.2.1 (Trapnell et al., 2010). Finally, all predicted genes from these three methods were merged and filtered by GLEAN v1.1 (Elsik et al., 2007) to create a consensus gene set.

Gene functional annotation of the sterlet genome was firstly performed by aligning all the protein sequences produced by GLEAN against public databases including Swiss-Prot, TeEMBL (Boeckmann et al., 2003) and KEGG (Kanehisa et al., 2016) using BLASTP v2.3.0+ (Altschul et al., 1990) with an e-value of 1e-5. Subsequently, motifs and domains were annotated using InterProScan (Hunter et al., 2008) by searching PANTHER (Thomas et al., 2003), Pfam (Finn et al., 2013), PRINTS (Attwood, 2002), ProDom (Bru et al., 2005) and SMART (Letunic et al., 2004) databases. Finally, InterProScan (Hunter et al., 2008) was applied to assign Gene Ontology (GO) terms and conduct a GO enrichment analysis (Ashburner et al., 2000).

### Fossil-Calibrated Phylogenetic Analysis

To perform a phylogenetic analysis of the sterlet, we obtained the predicted coding sequences (CDS) from the sterlet and 14 other vertebrates, including Asian arowana (*Scleropages formosus*), coelacanth (*Latimeria menadoensis*), common carp (*Cyprinus carpio*) and Atlantic salmon (*Salmo salar*) as well as the ten species used for homology gene annotation, and used the sea lamprey as the outgroup. BLAST with blastp mode and an e-value of 1e-5 were used to build the super similarity matrix, followed by OrthoMCL (Li et al., 2003) to distinguish gene families. One-to-one orthologues were identified by Markov Chain Clustering (MCL) and were aligned by MUSCLE v3.7 (Edgar, 2004). The first nucleotide of each codon was chosen to construct a Maximum-likelihood (ML) tree using PhyML v3.0 (Guindon et al., 2010) with gamma distribution across aligned sites and HKY85 substitution model. Branch supports were evaluated by approximate likelihood ratio test (aLRT). Meanwhile, we also conducted Bayesian inference (BI) independently using MrBayes v3.2.2 (Ronquist et al., 2012) to confirm the topology deduced from ML. Totally, we performed 100,000 generations and sampled every 100 generations. The initial 20% of the runs were regarded as unreliable samples and were discarded. The rest of the samples were used to estimate the branch supports. The divergence time of the sterlet from other vertebrates was estimated by Bayesian method using MCMCtree in PAML v4.9 (Yang, 2007) with two fossil calibrations, which are *Latimeria* (Sarcopterygii, 408.0 ∼ 427.9 Mya) and *Danio* (Teleostei, 151.2 ∼ 252.7 Mya; Hughes et al., 2018).

### 4dTv Analysis to Determine the Sturgeon-Specific Genome Duplication

We performed 4-fold degenerative third-codon transversion (4dTv) analysis to test the sturgeon-specific GD by comparing the sterlet genome to Asian arowana genome. Protein sequences from the two genomes were firstly aligned using all-to-all BLAST with blastp mode and an e-value of 1e-5. Subsequently, syntenic regions between sterlet-sterlet, arowana-arowana and sterlet-arowana were identified by MCscan v0.8 (Wang et al., 2012) with default parameters. Homologous protein sequences from these syntenic regions were retrieved and converted to CDS for alignment by MUSCLE (Edgar, 2004). Lastly, 4dTv values were calculated and corrected with the HKY model in PAML package (Yang, 2007).

### *Hox*-Cluster Identification and Phylogenetic Analysis

Reference protein sequences of complete *Hox*A cluster and partial *Hox*D cluster of American paddlefish (*Polyodon spathula*) (Crow et al., 2012) were downloaded from National Center of Biotechnology Information (NCBI). Sequences of four complete *Hox* clusters of the Indonesian coelacanth (Amemiya et al., 2010) and spotted gar (Braasch et al., 2015) were downloaded from Ensembl. The protein sequences were firstly aligned to the sterlet genome assembly by BLAST (Altschul et al., 1990) with tblastn mode and the hit sequences were further analyzed by Exonerate software (Slater and Birney, 2005) to extract exons. *Hox* gene order and synteny were finally determined by aligning back to the genome assembly and the best hits were selected by SOLAR (Yu et al., 2006). The *Hox*A clusters from the sterlet and paddlefish, as well as *HoxA9* genes from ten vertebrates were separately aligned with MEGA v7.0.26 (Kumar et al., 2016) followed by construction of a ML phylogenetic tree.

## Results and Discussion

### Summary of the Genome Sequencing and Assembly

We generated 316.8 Gb of pair-end raw reads (**Supplementary Table 1**) to assemble the draft genome of the sterlet. After filtering low-quality sequences, the data size of the remaining clean reads was about 248.4 Gb (**Supplementary Table 1**). The haploid genome size of the tarlet was estimated (**Supplementary Figure 2**) by a k-mer analysis (Li et al., 2010). Using all the clean reads, we produced a final genome assembly of 1.83Gb, which is quite close to the previously reported 1.87 Gb by flow cytometry (Birstein et al., 1993). The achieved draft assembly had a contig N50 of 18.88 kb and a scaffold N50 of 191.06 kb (Table 1).

**Table 1:** Statistics of assembled contigs and scaffolds.

Parameter	Contig		Scaffold	
	Size (bp)	Number	Size (bp)	Number
N90	450	257,242	1,325	38,164
N80	2,365	84,215	32,254	9,354
N70	7,919	48,548	62,161	5,330
N60	13,208	32,851	109,208	3,086
N50	18,882	22,595	191,062	1,801
Longest (bp)	223,430		5,122,172	
Total Size (bp)	1,622,894,949		1,831,554,666	
Total Number (>100 bp)	1,255,020		985,522	
Total Number (>2,000 bp)	91,019		27,173	

Accordingly, the genome sequencing depth for the tarlet reached 132-fold based on the final 1.83-Gb assembly, and as much as 87.19% of the bases had an over 20-fold sequencing depth (**Supplementary Figure 3**). The total completeness of the assembly was estimated to be 81.6% by evaluation with BUSCO, including 51.9% complete and single-copy BUSCOs and another 29.7% duplicated BUSCOs. A total of 4,584 genes were searched and 302 (6.6%) of them were fragmental BUSCOs (**Supplementary Table 2**). Along with the homogeneous GC distribution of the scaffolds (**Supplementary Figure 4**), we concluded that our draft assembly of the tarlet genome was qualified for further analyses.

### A Relatively High Content of Repetitive Elements

We performed repeat annotation, and a total of 784-Mb (42.84%) repeated sequences, including 726-Mb (39.68%) transposable elements (Tes) and 79 Mb (4.34%) tandem repeats, were identified in the tarlet genome assembly (**Supplementary Table 3**). These data are consistent with the dominant sub-peak ideally located at 2-fold the position of the main k-mer peak (**Supplementary Figure 2**). This repeat content was higher than those of the majority of the published fish genomes that usually contain no more than 40% repeats (Yuan et al., 2018). Interestingly, more class I (28.95%) than class II (14.93%) Tes were found in the tarlet genome (**Supplementary Table 4**), which resembled a cartilaginous species pattern (Yuan et al., 2018). In addition, as a potamodromous species dwelling mainly in freshwater, the sterlet had a relatively high DNA/TcMar-Tc1 proportion (16.58% for 130 Mb) but a relatively low microsatellites proportion (2.10% for 16 Mb) (**Supplementary Table 5**), a pattern preferred by freshwater species (**Supplementary Figure 5**; Yuan et al., 2018).

Furthermore, we identified 318 copies of *Tana1*, a new putative active *Tc1*-like transposable element (Pujolar et al., 2013) but not referred in the repeat annotation library (Romanenko et al., 2015). Our results showed that 299 of the predicted *Tana1* copies contain full-length transposases. Interestingly, the majority of these *Tana1* copies did not have internal stop codon(s) as determined in the a previous study (Pujolar et al., 2013), suggesting that this element is more likely to be active. The 299 complete *Tana1* genes were from 250 different scaffolds, with an average of 1.19 genes in each scaffold. Sequences and gene locations of the identified *Tana1* are publicly available in figshare with an accession ID of doi: 10.6084/m9.figshare.8289881.

We then calculated the number of repeats that were co-localized with the protein coding genes after gene annotation to estimate their potential functions. Our results showed that a total of 34,987 repeats (14.23 Mb in length, accounting for 1.82% of all repeats) were co-localized with 10,460 protein coding genes, among which LINE/CR1, DNA/TcMar-Tc1 and LINE/L2 were the most abundant types (**Supplementary Data Sheet 2**). The GO enrichment analysis revealed that these repeats were enriched into 52 terms. Cellular process, binding, single-organism process, metabolic process and biological regulation were the top five enriched ones (**Supplementary Figure 6**), indicating that these repeats may participate in such biological processes.

However, the distribution and location of these repeats and annotated genes on chromosomes are still awaiting identification with assistance of on-going PacBio sequencing. It seems that repetitive DNA sequences have a tendency to cluster in specific regions, such as in pericentromeric, centromeric and telomeric regions (Biltueva et al., 2017). The potential roles of repetitive sequences in chromosomal rearrangements will also be much clearer, once a chromosome-level genome assembly is available for the sterlet.

### Statistics of Gene Annotation and Phylogenetic Analysis

After masking the abundant repeats in scaffolds, we annotated 22,184 protein-coding genes with an average gene length of 21 kb using a combined strategy of *ab initio*, homology-based and transcriptome-based annotation. This predicted gene number of the sterlet genome seems to be lower than estimation, possibly due to missing data and many gaps in the draft assembly. In addition, the repetitive sequences and complex polyploidy (Romanenko et al., 2015) make it more difficult to produce a fine assembly and to predict a complete gene set. Our BUSCO analysis of the gene set showed that complete and fragmented BUSCOs accounted for 73.2% of the searched genes, and 26.8% were missing BUSCOs (**Supplementary Table 2**); we therefore inferr that the total gene number of the sterlet could reach 28,136 (with the addition of the missing BUSCOs), which is more than that of a diploid fish but less than a tetraploid species when taking the partial tetraploidy into consideration. Statistics of the gene list are provided in **Supplementary Table 6**. Length distributions of the predicted genes, CDS, exons and introns were comparable to those of spotted gar, elephant shark and many other fishes (**Supplementary Figure 7**). Of all these genes, a total of 21,112 genes (95.17%) were functionally annotated in at least one public database (find more details in **Supplementary Table 7**).

**Figure 1:**
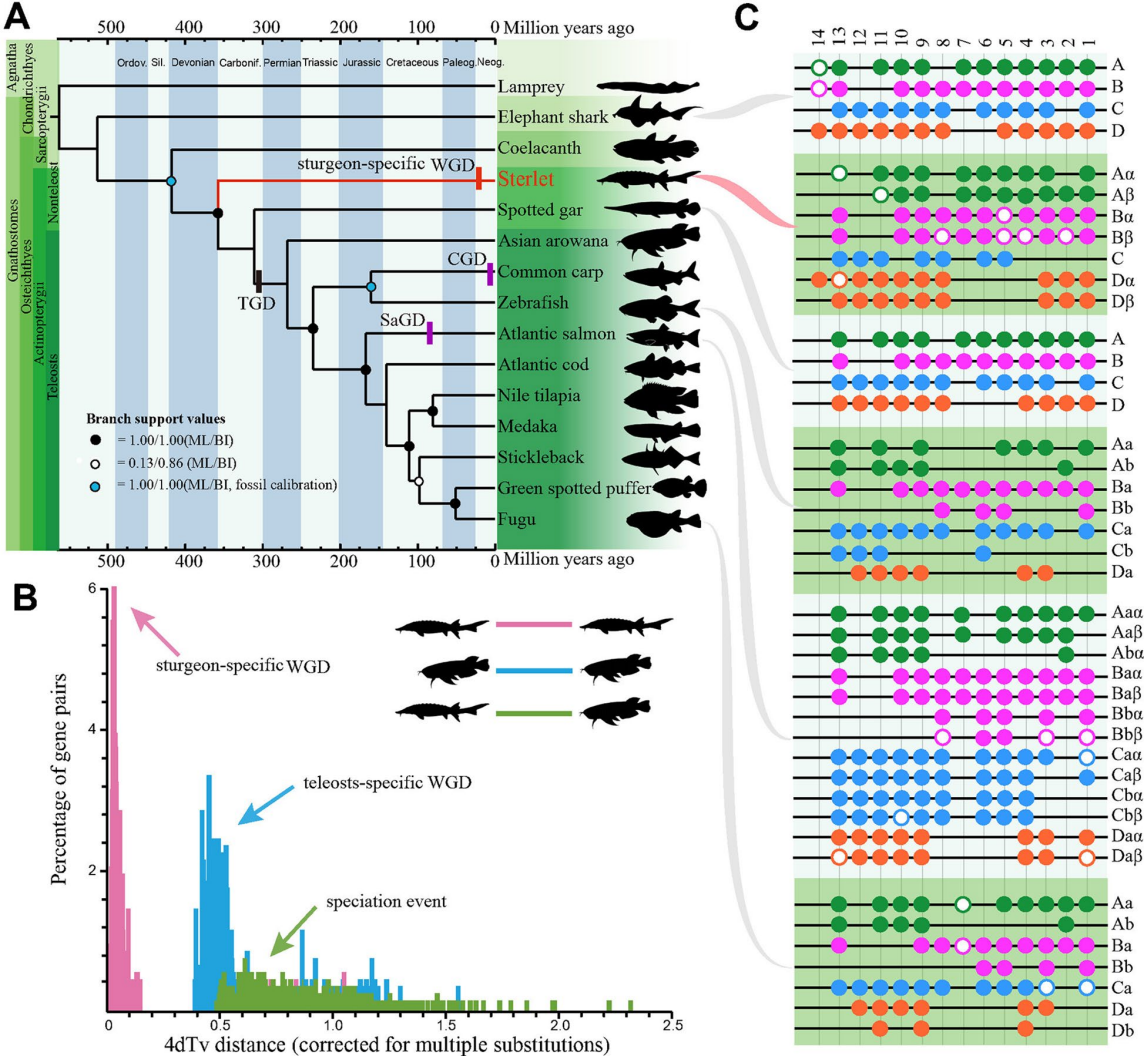
The sterlet takes the most basal position at the phylogeny of ray-finned fish and evolved seven *Hox* clusters after the lineage-specific whole genome duplication. **(A)** The fossil-calibrated phylogenetic tree of 15 examined vertebrates including the sterlet. The phylogenetic topology was deduced by both the ML and BI methods. TGD, teleost-specific GD; CGD, carp GD; SaGD, salmonid GD. **(B)** A 4dTv comparison between Asian arowana and the sterlet. **(C)** Presence of *Hox* clusters in elephant shark (Venkatesh et al., 2014), sterlet (this study), spotted gar (Braasch et al., 2015), zebrafish (Bian et al., 2016), Atlantic salmon (Lien et al., 2016) and fugu (Bian et al., 2016). Each black line refers to a *Hox* cluster. Solid circles represent complete *Hox*A (green), *Hox*B (pink), *Hox*C (blue) and *Hox*D (orange) genes, while hollow circles stand for pseudo or partial genes. Paralogs generated by TGD were labeled with a and b, whereas paralogs produced by lineage-specific GD were named by α and β.

Afterwards, the predicted CDS sequences along with whole-genome CDS from other 14 examined vertebrates were clustered into gene families to determine 198 single-copy consensus orthologues from these genomes (**Supplementary Table 8**; **Supplementary Figure 8**), which were selected out for generation of the phylogenetic topology by ML (**Supplementary Figure 9**) or BI (**Supplementary Figure 10**). The two methods produced a complete coincidence of phylogenetic topology with high branch support values, suggesting that the hypothesis was well supported (**Figure 1A**). Our tree confirms the results of others (Hughes et al., 2018; Peng et al., 2007), that the sterlet is located at a base position of Actinopterygii, which serves as a sister group to all ray-finned fishes. Therefore, this phylogeny of the sterlet using numerous single-copy genes confirms its very basal position as reported by other studies. Fossil calibrations date the origin of the sterlet back to 358 Mya (**Figure 1A**), with a 95% confidence interval of 316∼394 Mya (**Supplementary Figure 11**). These data are consistent with our previous comprehensive phylogeny analysis (Hughes et al., 2018), and most interestingly, this date is extremely close to the Late Devonian Extinction that happened around 358.9 Mya (McGhee et al., 1984).

### Identification of an Independent WGD Event that Occurred Recently in the Sterlet

Sturgeons didn’t experience the TGD event (Ravi and Venkatesh, 2018), but there are clear evidences that there was a sturgeon-specific GD event (Havelka et al., 2016). In order to identify this lineage-specific GD in the sterlet, we performed a 4dTv analysis along with Asian arowana (Bian et al., 2016), which had experienced the TGD event around 320 Mya (Jaillon et al., 2004). Our analysis displayed distinct peaks in each group of sterlet-sterlet (sturgeon-specific GD), arowana-arowana (TGD) and sterlet-arowana (speciation event), and the synonymous transversions rates (Ks values) were estimated to be 0.03 and 0.45 in the sterlet and Asia arowana, respectively (**Figure 1B**). Hence, the sturgeon-specific GD was estimated to have occurred about 21.3 Mya ([320 Mya/0.45]*0.03) d, long after the evolutionary splitting between the sturgeon and paddlefish (184 Mya; Peng et al., 2007). Hence, it that sturgeons (Acipenseridae) and paddlefish (Polyodontidae) experienced polyploidization events independently.

### Characterization of the Complete *Hox* Clusters

To provide additional insights into polyploidy of the genome at the gene level after the sturgeon-specific GD event, we investigated *Hox* gene clusters in the sterlet genome. We identified seven *Hox* clusters including 68 *Hox* genes (60 intact and 8 partial/pseudo genes) in the draft assembly (**Figure 1C**, **Supplementary Data Sheet 3**). The *Hox* data seemed to be a consequence of the sturgeon-specific GD, since only four *Hox* clusters were identified in sea lamprey (43 genes), elephant shark (47 genes) and spotted gar (43 genes; Venkatesh et al., 2014). Interestingly, the possible absence of a whole *Hox*C cluster in the sterlet is similar to that in some diploid teleost such as fugu, medaka and stickleback (Pascual-Anaya et al., 2013). Furthermore, our *Hox*A based genealogy showed that, contrary to the *Hox* pattern in teleost after TGD (**Supplementary Figure 12**), *Hox*A clusters from the sterlet and paddlefish formed two separate groups (**Supplementary Figure 12**), which indicates that *Hox* genes duplicated independently after the divergence of the two families. It confirmed the independence of lineage-specific GDs in the sterlet and paddlefish, which is consistent with our above-mentioned prediction by 4dTv.

However, whether this WGD is sturgeon-specific or shared by all members of the Acipenseridae family is awaiting answers from genome sequencing of more sturgeon species. Furthermore, the present research on a complete gene-chromosome pattern of the sterlet genome is still preliminary, but this work and a previous report of sequencing 15 chromosome-specific libraries (Andreyushkova et al., 2017) provide some novel insights. We attempted to map our assembly to the spotted gar chromosomes, but the results were difficult to interpret, possibly due to the non-full-length assembly of our current draft genome, the great complexity of the sterlet chromosomes, and high sequence divergences between the two fish species. Therefore, based on our current knowledge on the sterlet genome (Romanenko et al., 2015; Andreyushkova et al., 2017), a chromosome-level assembly needs to be generated, with assistance of long-read sequencing and chromatin conformation capture technology for a better understanding of the complicated structure and evolutionary pattern of the sterlet genome.

## Data Availability

The datasets generated for this study can be found in the NCBI with accession PRJNA491785, SRR8371834 ∼ SRR837184.

## Ethics Statement

All experiments in the present study were carried out in accordance with the guidelines of the Animal Ethics Committee of Yangtze River Fisheries Research Institute of Chinese Academy of Fishery Sciences (No. YFI-01).

## Author Contributions

QW, HD, CL, JX, and QS, conceived and designed the project. YH, PC, YL, and CB, analyzed the data. CL, RR, HY, and XY collected and processed the samples. PC, YH, and QS wrote the manuscript. QS, XL and QW revised the manuscript. All authors have read and approved the final manuscript and declared no competing interests.

## Funding

The study was supported by the the National Natural Science Foundation of China (grant number NSFC 31772854), China Postdoctoral Science Foundation (grant number 2017M622560), the National Program on Key Basic Research Project (973 Program, 2015CB15072), Hubei Postdoctoral Innovation Post Project (No. 2017C08), Shenzhen Special Program for Development of Emerging Strategic Industries (No. JSGG20170412153411369) and Office of Fisheries Supervision and Management for the Yangtze River Basin, MARA, PRC (No. 171821301354051046).

## Conflict of Interest Statement

The authors declare that the research was conducted in the absence of any commercial or financial relationships that could be construed as a potential conflict of interest.

The reviewer JM declared a shared affiliation, with no collaboration, with several of the authors, PC, XL, to the handling editor at the time of review.
