# Supplementary material for: Draft Genome and Complete Hox-Cluster Characterization of the Sterlet (Acipenser ruthenus)
Source: Front Genet. 2019 Sep 5;10:776. doi: 10.3389/fgene.2019.00776 (PMC6739705; doi:10.3389/fgene.2019.00776)
Supplement: Supplementary file 1 [file DataSheet_1.docx]

Supplementary Materials

Draft Genome and Complete *Hox*-Cluster Characterization of the Sterlet (*Acipenser ruthenus*)

Peilin Cheng ^1,2^†, Yu Huang ^1,3,4^†, Hao Du ^1^, Chuangju Li ^1^, Yunyun Lv ^3,4^, Rui Ruan ^1^, Huan Ye ^1^, Chao Bian ^3^, Xinxin You ^3^, Junmin Xu ^3,5^, Xufang Liang ^2^, Qiong Shi ^3,4^*, Qiwei Wei ^1^*

^1^ Key Laboratory of Freshwater Biodiversity Conservation, Ministry of Agriculture of China, Yangtze River Fisheries Research Institute, Chinese Academy of Fishery Sciences, Wuhan 430223, China

^2^ College of Fisheries, Chinese Perch Research Center, Huazhong Agricultural University, 430070, China

^3^ Shenzhen Key Lab of Marine Genomics, Guangdong Provincial Key Lab of Molecular Breeding in Marine Economic Animals, BGI Academy of Marine Sciences, BGI Marine, BGI, Shenzhen 518083, China

^4^ BGI Education Center, University of Chinese Academy of Sciences, Shenzhen 518083, China

^5^ School of Veterinary Medicine, Rakuno Gakuen University, Ebetsu 069-8501, Japan

**†Contributed equally to this project.**

*** Correspondence:**

Qiwei Wei

weiqw@yfi.ac.cn
Qiong Shi
shiqiong@genomics.cn

Supplementary Figures 1~12

Supplementary Tables 1~10

# Supplementary Figures and Tables

## Supplementary Figures

**Supplementary Figure 1.** A flow chart of sequencing, assembly and annotation process for the sterlet genome analysis.


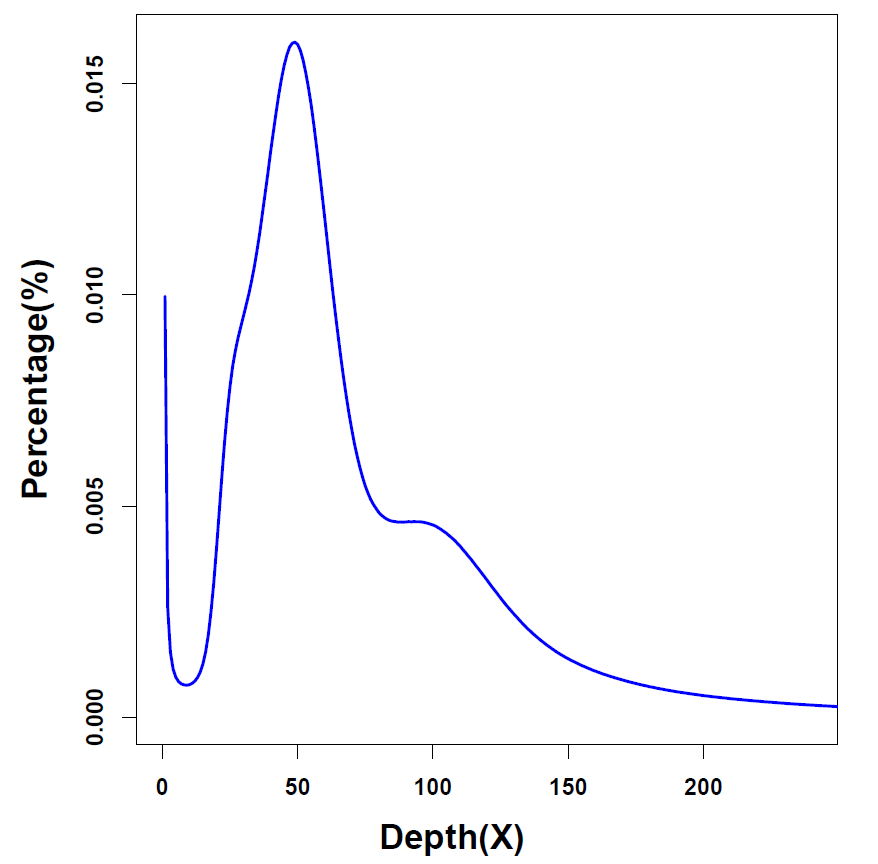


**Supplementary Figure 2. A** K-mer (k = 17) analysis to estimate the sterlet genome size. The first main peak (x = 49) is used for estimation of the genome size with the following formula: Genome size = k-mer num/peak depth. Certain repeats caused the sub-peak at 2-fold position of the main peak (x= 98).


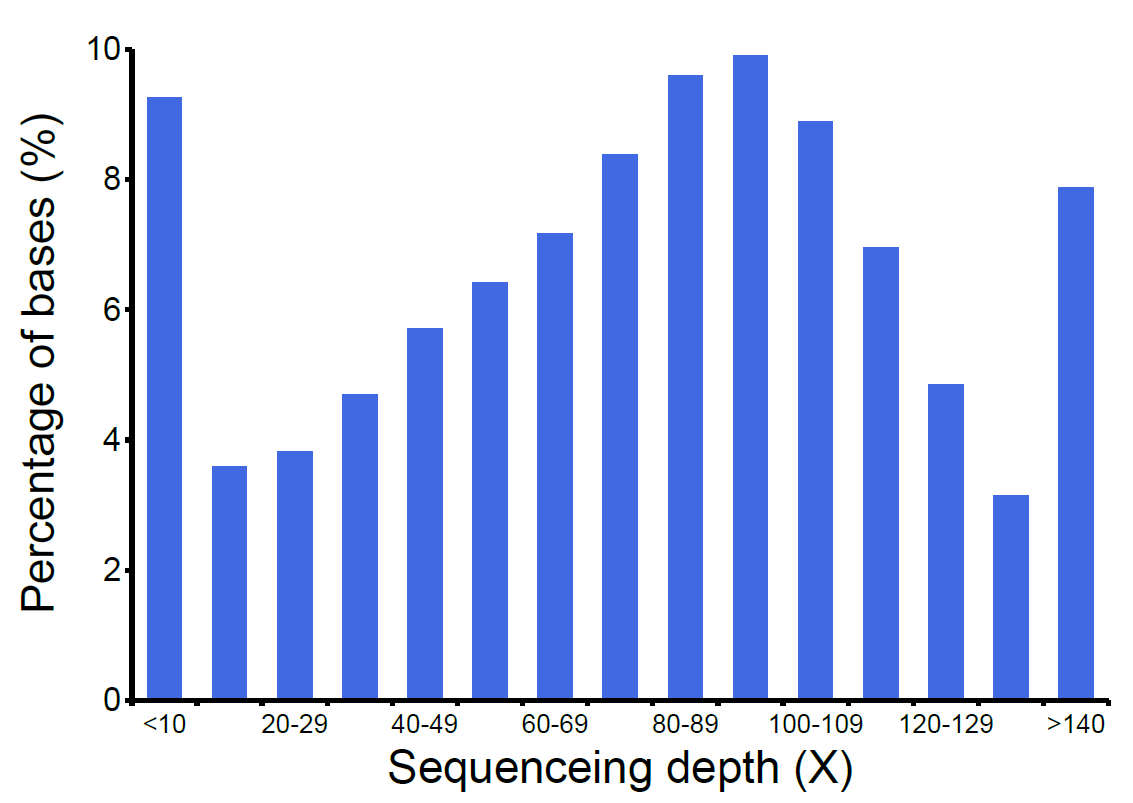


**Supplementary Figure 3.** Sequence depth distribution of the clean reads. The X-axis represents sequence depths, and the Y-axis represents the percentage of base numbers at a given depth.


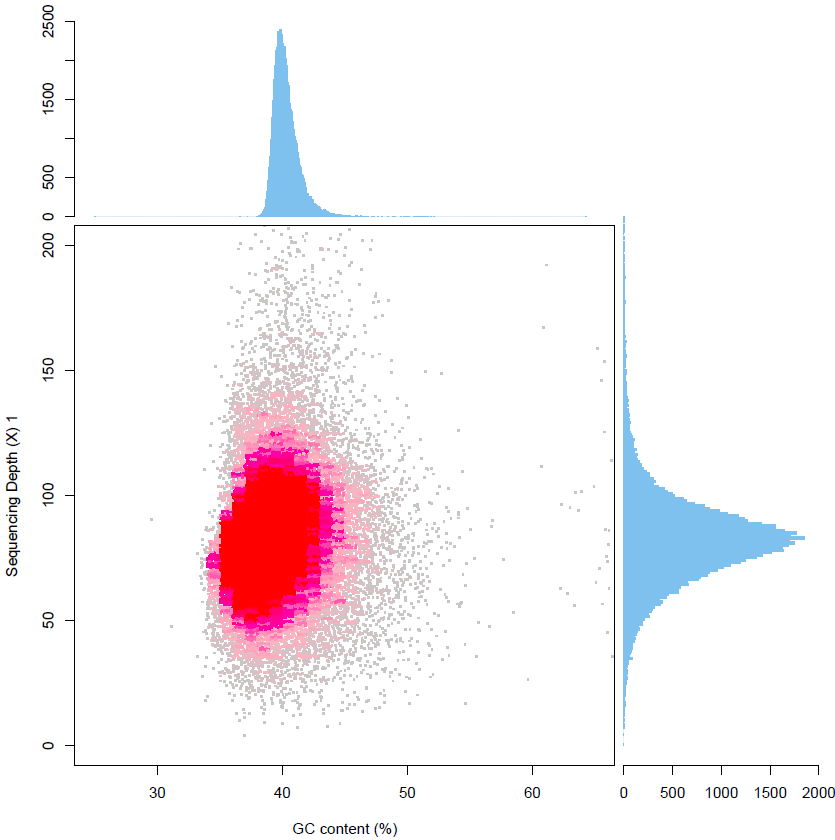


**Supplementary Figure 4.** GC content distribution and sequencing depth of the sterlet draft assembly. The main scatter plot was drawn by sliding 50-kb non-overlapping windows against the assembly. The X-axis represents the proportion of GC content, and the Y-axis represents the average sequencing depth. The upper and right bar charts show the numbers of hits at the given GC content or sequencing depth, respectively.


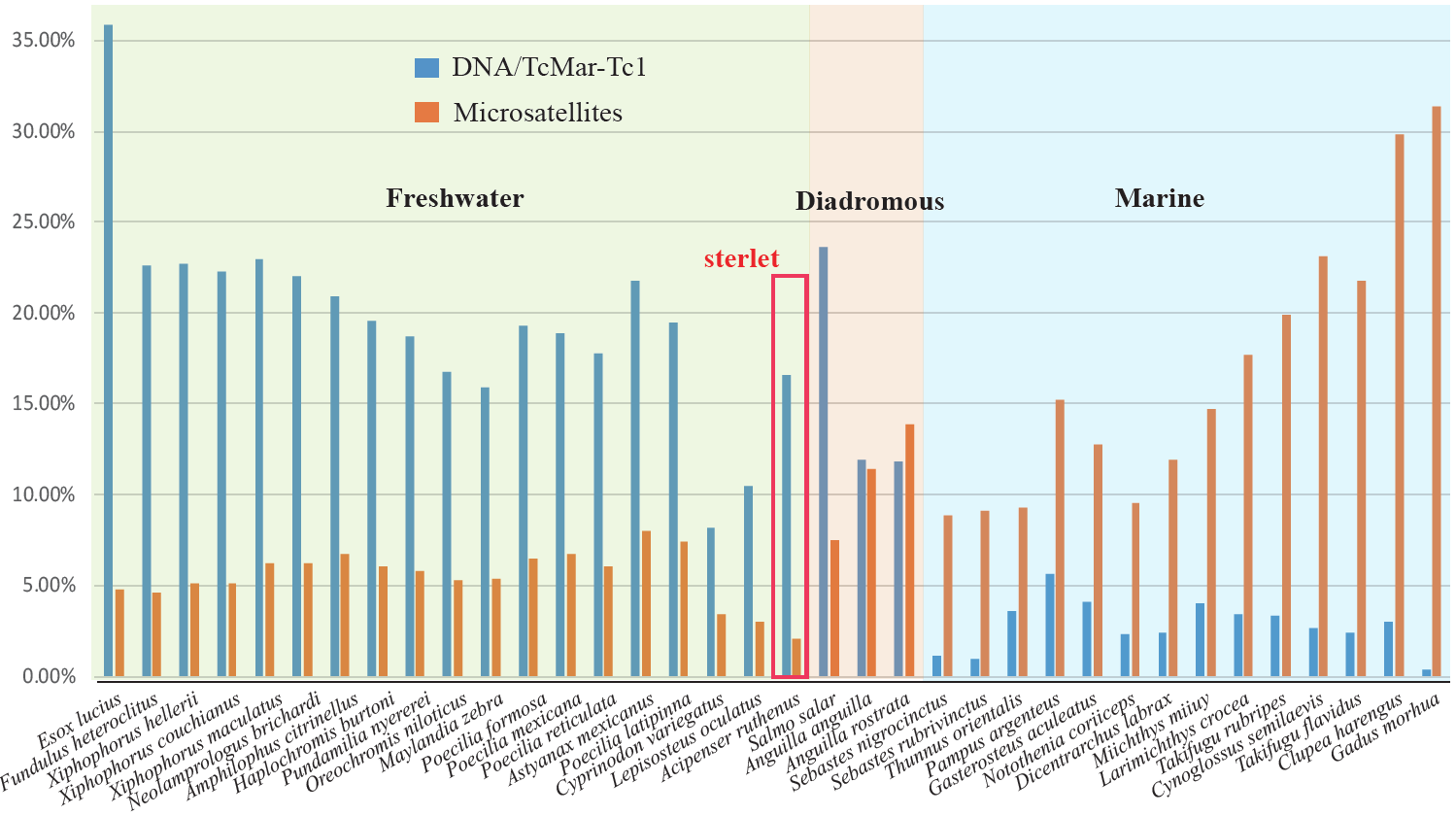


**Supplementary Figure 5.** Contents and distributions of DNA/TcMar-Tc1 and microsatellites in the genomes of sterlet and other examined fishes. The freshwater fishes are presented on the left (light yellow background) and marine species are on the right (light blue background), and diadromous ones are in the middle (light orange background). Blue bar represents DNA/TcMar-Tc1content (%) and orange bar stands for the proportion of the microsatellites (%).

**Supplementary Figure 6.** GO enrichment of the repetitive sequences that are co-localized with protein coding genes. A total of 52 terms were enriched into three categories, biological process (blue), cellular component (red) and molecular function (green). The right Y-axis shows the number of genes enriched in a certain term and the left Y-axis present the percentage.


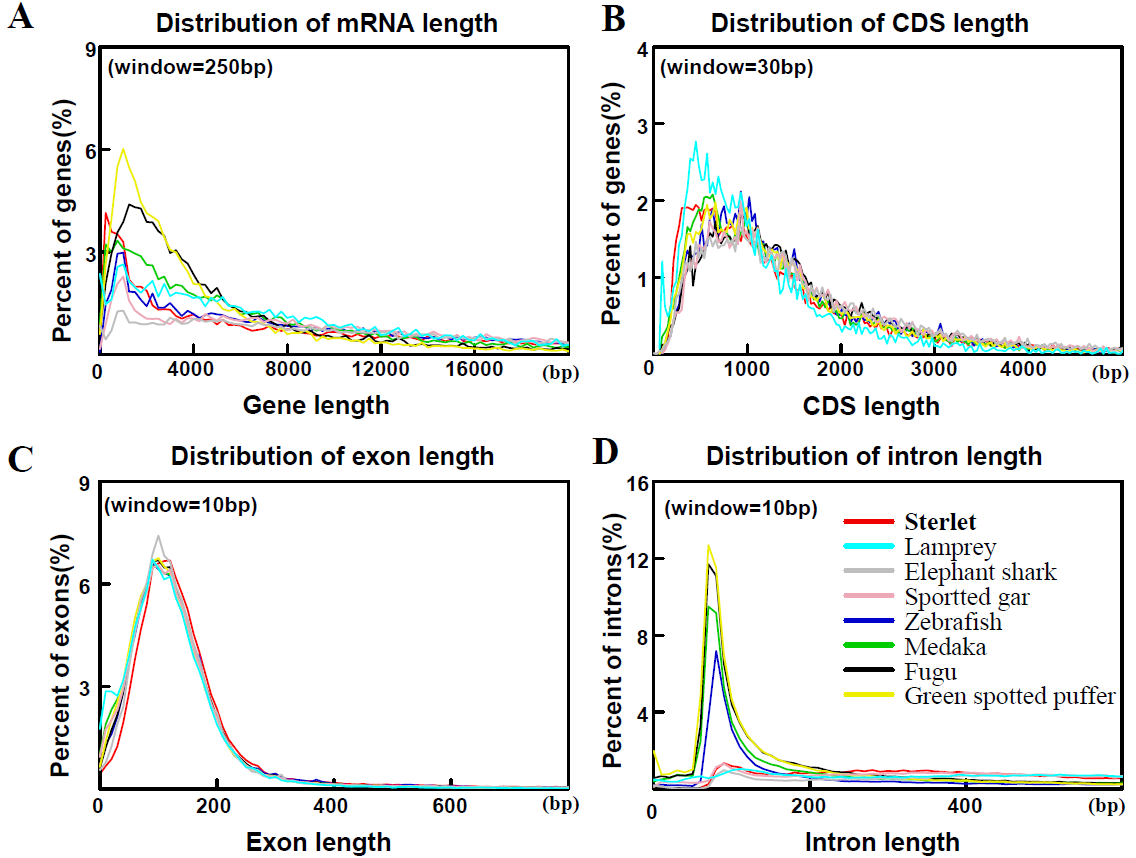


**Supplementary Figure 7.** Comparison of gene sets between the sterlet and other vertebrates. The four line plots show the length distribution of genes (A), CDS (B), exons (C) and introns (D). Each line with different color represents a special species. Please see more details of the species names in the pilot D.


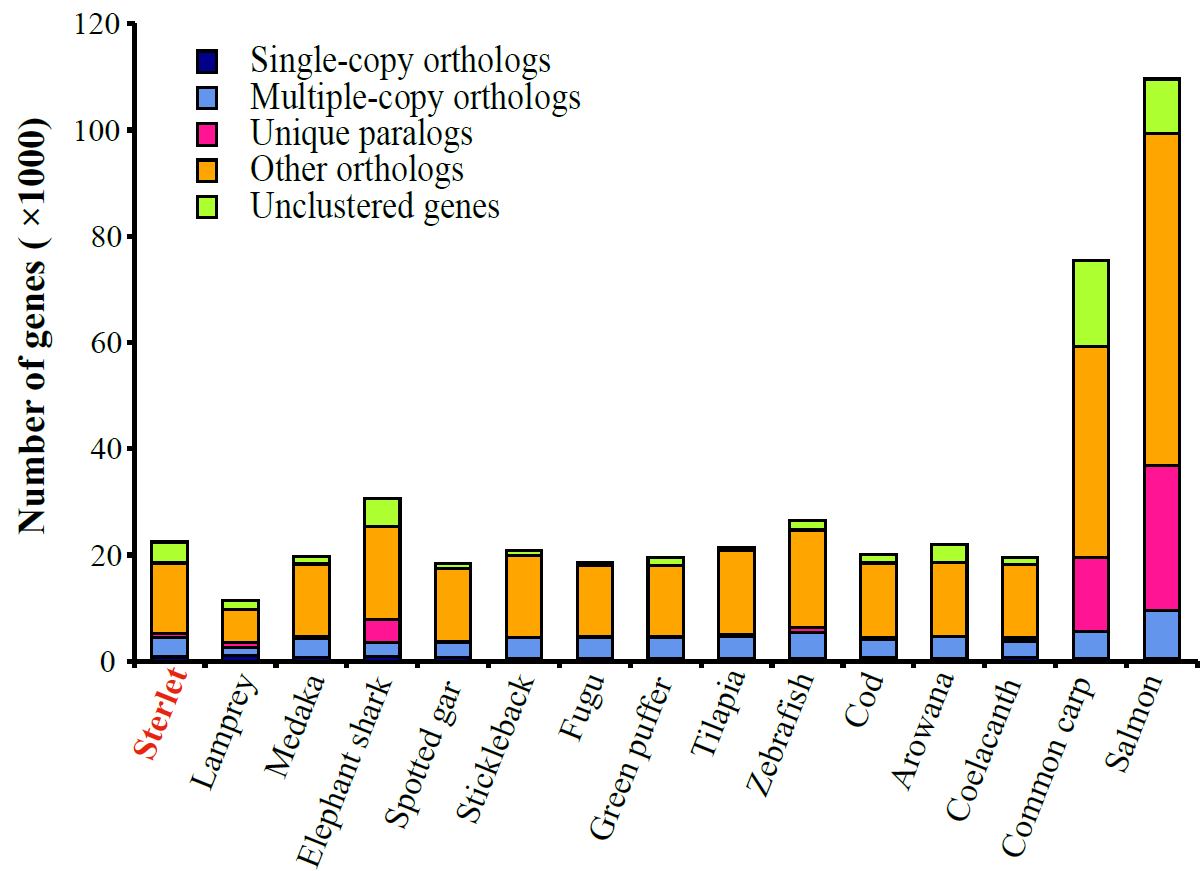


**Supplementary Figure 8.** Gene number distribution in each type of cluster between the sterlet and other vertebrates. The X-axis shows the common name of the examined vertebrates, and the Y-axis represents the number of genes (* 1000).


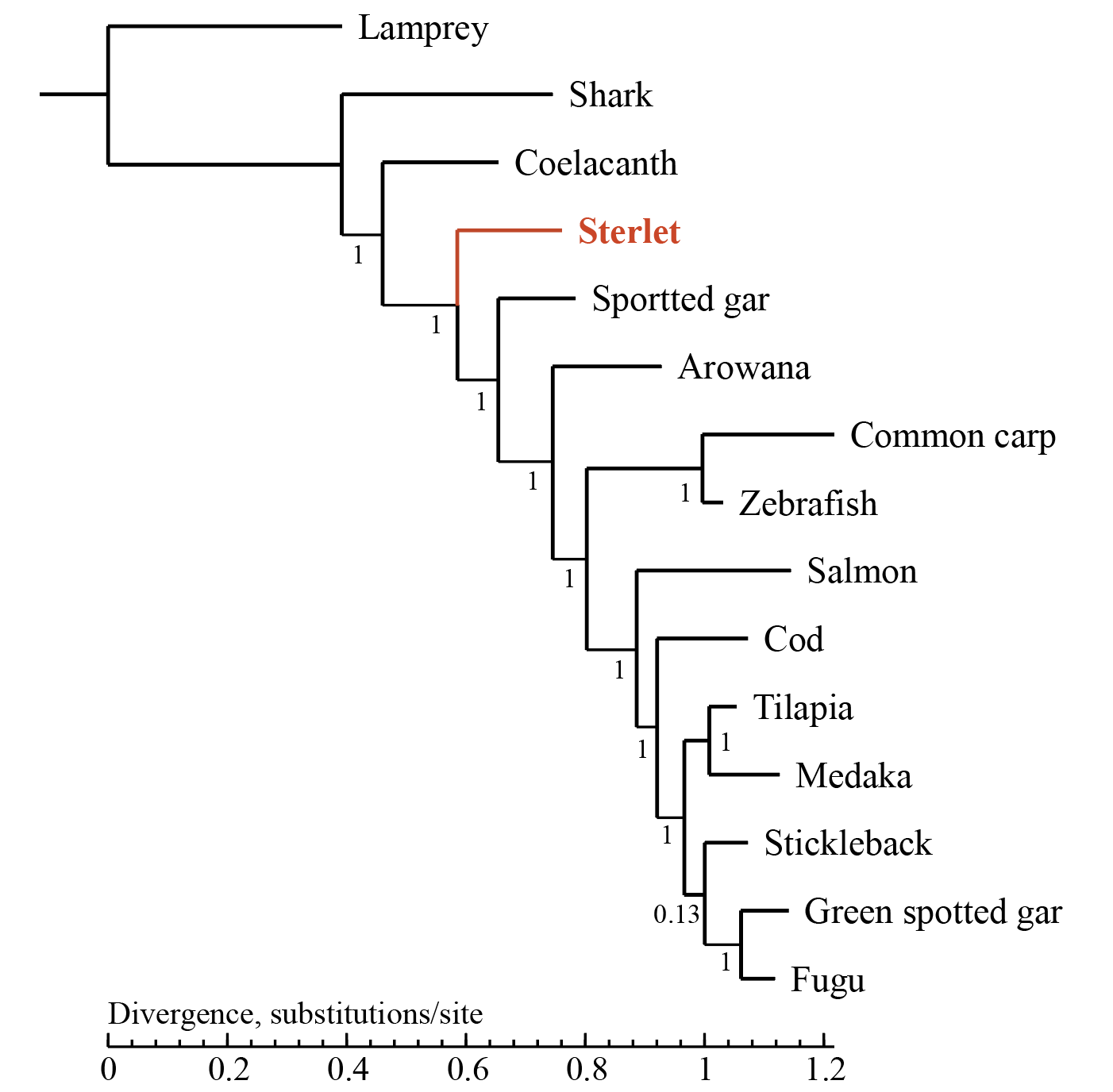


**Supplementary Figure 9. A** phylogenetic tree based on the Maximum likelihood (ML) method using PhyML with gamma distribution across aligned sites and HKY85 substitution model. Branch supports were evaluated by the approximate likelihood ratio test (aLRT).


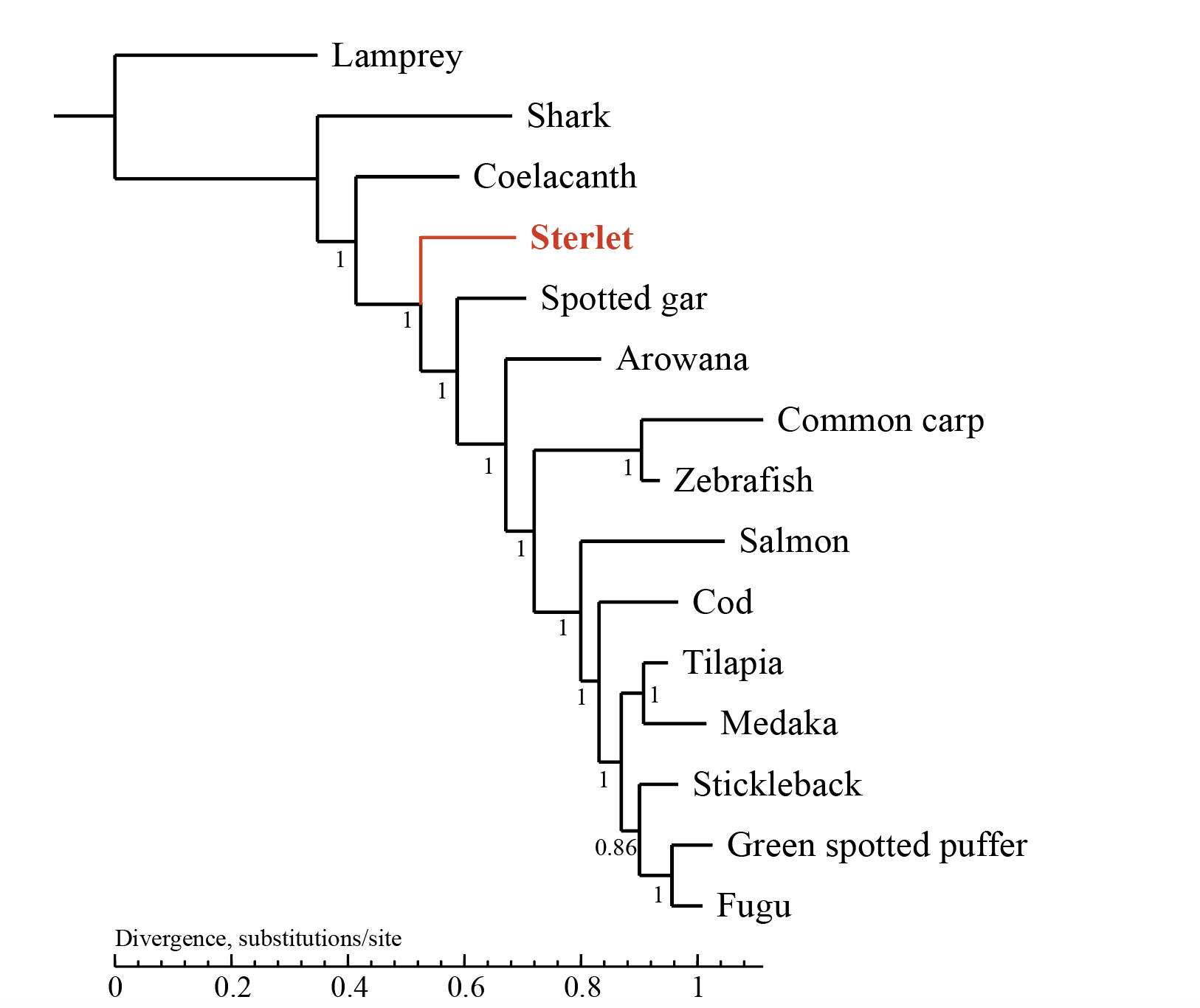


**Supplementary Figure 10. A** phylogenetic tree based on the Bayesian inference (BI) method using MrBayes. A total of 100,000 generations were performed, and every 100 generations were sampled. The initial 20% of the runs were burned, and the rest were used to estimate the branch supports.


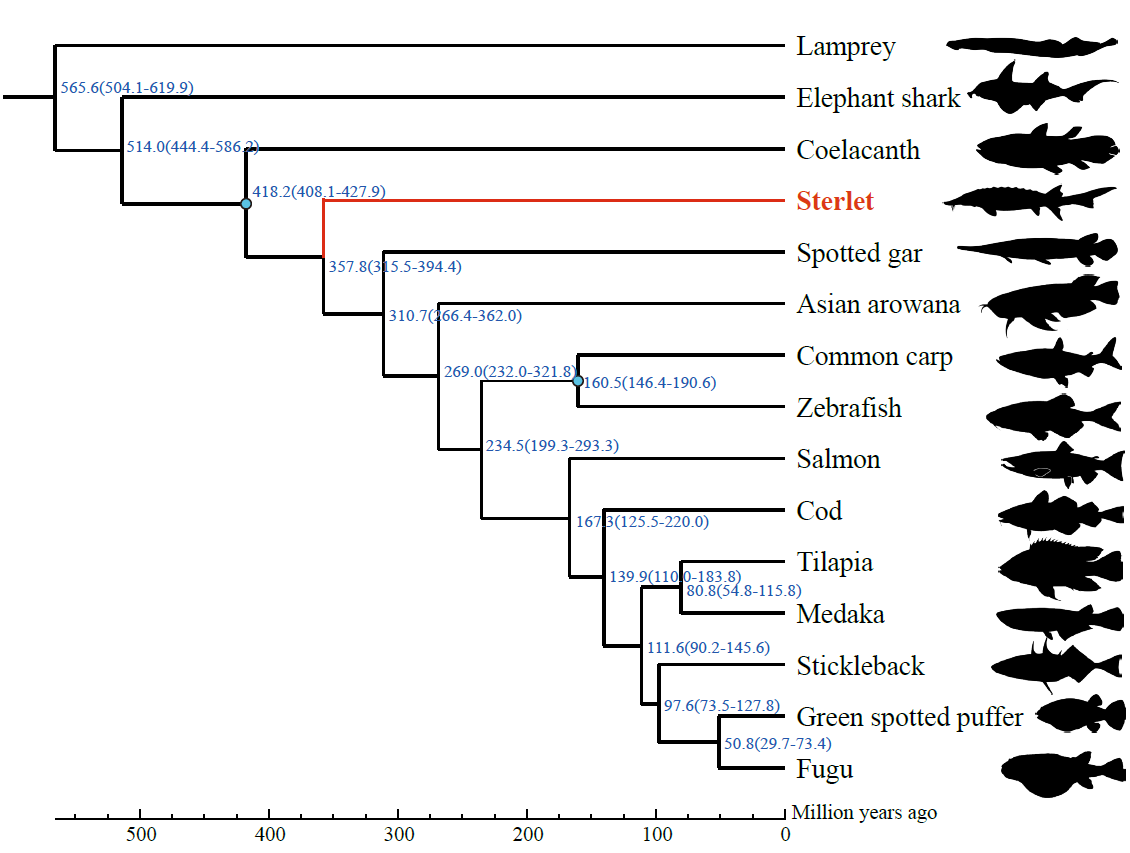


**Supplementary Figure 11.** Divergence time estimation with confidence intervals among the sterlet and other vertebrates on the basis of fossils calibration by Beyesian method using MCMC tree in PAML. The two blue circles represent the used calibrations.

**Supplementary Figure 12. A** phylogenetic tree of *Hox*A9 protein sequences from sterlet, paddlefish and other vertebrates. The small window in the the upper right corner shows the topology of evolutionary tree of *Hox*A clusters from sterlet and paddlefish, using protein sequences of all complete *Hox*A genes (*Hox*A1, *Hox*A3, *Hox*A4, *Hox*A5, *Hox*A6, *Hox*A9 and *Hox*A10) from both species. TGD, teleost-specific genome duplication (GD); SaGD, salmonid GD; CGD, carp GD.

## Supplementary Tables

**Supplementary Table 1.** Sequencing statistics of raw reads and clean data used to assemble the sterlet genome.

| **Data type** | **Insert Size**  **(bp)** | **Read Length**  **(bp)** | **Data Size**  **(Gb)** | **Sequencing coverage**^*^  **(X)** |
| --- | --- | --- | --- | --- |
| Raw data | 270 | 150 | 135.4 | 73.99 |
|  | 500 | 125 | 35.0 | 19.13 |
|  | 800 | 125 | 18.2 | 9.95 |
|  | 2,000 | 125 | 32.7 | 17.87 |
|  | 5,000 | 125 | 31.5 | 17.21 |
|  | 10,000 | 125 | 33.8 | 18.47 |
|  | 20,000 | 125 | 30.2 | 16.50 |
| Total |  |  | 316.8 | 173.11 |
| Clean data | 270 | 145 | 125.3 | 68.48 |
|  | 500 | 120 | 30.9 | 16.87 |
|  | 800 | 120 | 16.7 | 9.12 |
|  | 2,000 | 120 | 20.1 | 10.99 |
|  | 5,000 | 120 | 19.0 | 10.40 |
|  | 10,000 | 120 | 20.7 | 11.33 |
|  | 20,000 | 120 | 15.7 | 8.60 |
| Total |  |  | 248.4 | 135.74 |

*Sequence coverage was calculated based on the assembled genome with a size of 1.83 Gb.

**Supplementary Table 2.** BUSCO analysis to estimate the completeness of the assembly and gene set.

| **Type of BUSCOs** | **Assembly** | | **Gene set** | |
| --- | --- | --- | --- | --- |
|  | **Number of sequences** | **Ratio** | **Number of sequences** | **Ratio** |
| **Complete** | 3741 | 81.6% | 2807 | 61.2% |
| **Complete and single-copy** | 2381 | 51.9% | 1835 | 40.0% |
| **Complete and duplicated** | 1360 | 29.7% | 972 | 21.2% |
| **Fragmented** | 302 | 6.6% | 547 | 11.9% |
| **Missing** | 541 | 11.8% | 1230 | 26.8% |
| **Total** | 4584 | 100% | 4584 | 100% |

**Supplementary Table 3.** Summary of repeats annotation in the sterlet genome.

| **Type** | **Method** | **Repeat Size (bp)** | **% of Genome** | **% of Repeats** |
| --- | --- | --- | --- | --- |
| Tandem Repeats | TRF | 79,578,249 | 4.34 | 10.14 |
| Transposable elements  (TEs) | Repeatmasker | 134,869,304 | 7.36 | 17.19 |
|  | Proteinmask | 95,031,991 | 5.18 | 12.11 |
|  | *De novo* | 633,176,474 | 34.57 | 80.71 |
| Total |  | 784,555,134 | 42.84 | 100% |

**Supplementary Table 4.** Statistics of transposable elements (TEs) in the sterlet genome.

| **Class** | **Type** | **Repbase TEs^*^** | | **TE proteins^*^** | | ***De novo^*^*** | | **Combined TEs** | |
| --- | --- | --- | --- | --- | --- | --- | --- | --- | --- |
|  |  | **Length (bp)** | **%^**^** | **Length (bp)** | **%^**^** | **Length (bp)** | **%^**^** | **Length (bp)** | **%^**^** |
| I | LINE | 44,640,283 | 2.44 | 45,103,653 | 2.47 | 210,652,768 | 11.50 | 271,724,817 | 14.84 |
| I | SINE | 8,890,409 | 0.49 | 0 | 0 | 66,947,153 | 3.66 | 73,652,233 | 4.02 |
| I | LTR | 26,479,088 | 1.45 | 22,771,386 | 1.24 | 149,870,111 | 8.18 | 184,795,829 | 10.09 |
| II | DNA | 64,499,850 | 3.52 | 27,239,537 | 1.49 | 216,739,332 | 11.83 | 273,408,187 | 14.93 |
| - | Other | 190,970 | 0.01 | 0 | 0 | 177,373 | 0.01 | 368,133 | 0.02 |
| - | Unknown | 0 | 0 | 0 | 0 | 21,135,026 | 1.15 | 21,135,026 | 1.15 |
| - | Total | 134,869,304 | 7.36 | 95,031,991 | 5.19 | 611,537,665 | 33.39 | 726,672,696 | 39.68 |

**Supplementary Table 5.** Subtypes of transposable elements (TEs) and other repeats in the sterlet genome.

| **Subtype** | **Length** | **%^*^** | **Subtype** | **Length** | **%^*^** |
| --- | --- | --- | --- | --- | --- |
| LINE/Ambal | 43,118 | 0.005 | DNA/Academ | 747,488 | 0.095 |
| LINE/CR1 | 157,964,180 | 20.134 | DNA/Academ2 | 9,035 | 0.001 |
| LINE/CR1-Zenon | 233,863 | 0.030 | DNA/CMC-Chapaev | 2,727,190 | 0.348 |
| LINE/CRE | 1,305 | 0.000 | DNA/CMC-Chapaev-3 | 25,682,562 | 3.274 |
| LINE/CRE-Cnl1 | 366 | 0.000 | DNA/CMC-EnSpm | 18,526,426 | 2.361 |
| LINE/DRE | 297,541 | 0.038 | DNA/CMC-Mirage | 17,047 | 0.002 |
| LINE/Dong-R4 | 129,048 | 0.016 | DNA/CMC-Transib | 433,473 | 0.055 |
| LINE/Genie | 357 | 0.000 | DNA/Crypton | 2,624,371 | 0.335 |
| LINE/I | 474,830 | 0.061 | DNA/Crypton-H | 1,724 | 0.000 |
| LINE/I-Nimb | 193,715 | 0.025 | DNA/Crypton-V | 788,870 | 0.101 |
| LINE/Jockey | 1,937,033 | 0.247 | DNA/DNA | 39,425,273 | 5.025 |
| LINE/L1 | 9,935,614 | 1.266 | DNA/Dada | 643,781 | 0.082 |
| LINE/L1-Tx1 | 9,073,367 | 1.156 | DNA/Ginger | 4,263,309 | 0.543 |
| LINE/L2 | 67,271,532 | 8.574 | DNA/Harbinger | 159,394 | 0.020 |
| LINE/L2-Hydra | 1,103 | 0.000 | DNA/Helitron | 5,302,386 | 0.676 |
| LINE/LINE | 18,583,346 | 2.369 | DNA/IS | 144 | 0.000 |
| LINE/LOA | 36,274 | 0.005 | DNA/IS3EU | 696,517 | 0.089 |
| LINE/Odin | 222 | 0.000 | DNA/Kolobok | 10,294 | 0.001 |
| LINE/Penelope | 4,222,000 | 0.538 | DNA/Kolobok-Hyd | 498 | 0.000 |
| LINE/Proto1 | 368,475 | 0.047 | DNA/Kolobok-Hydra | 520,539 | 0.066 |
| LINE/Proto2 | 3,044 | 0.000 | DNA/Kolobok-T2 | 473,890 | 0.060 |
| LINE/R1 | 689,709 | 0.088 | DNA/MULE-F | 13,551 | 0.002 |
| LINE/R2 | 1,145,261 | 0.146 | DNA/MULE-MuDR | 1,599,428 | 0.204 |
| LINE/R2-Dualen | 471 | 0.000 | DNA/MULE-NOF | 6,908 | 0.001 |
| LINE/R2-Hero | 14,551,354 | 1.855 | DNA/Maverick | 3,768,655 | 0.480 |
| LINE/R2-NeSL | 4,513 | 0.001 | DNA/Merlin | 521,177 | 0.066 |
| LINE/RTE | 17,350 | 0.002 | DNA/MuLE-F | 99 | 0.000 |
| LINE/RTE-BovB | 1,489,570 | 0.190 | DNA/MuLE-MuDR | 50,781 | 0.006 |
| LINE/RTE-RTE | 761,659 | 0.097 | DNA/MuLE-NOF | 77,467 | 0.010 |
| LINE/RTE-RTEX | 206,418 | 0.026 | DNA/Novosib | 5,955,570 | 0.759 |
| LINE/RTE-X | 3,454,213 | 0.440 | DNA/P | 892,510 | 0.114 |
| LINE/Rex-Babar | 1,296,362 | 0.165 | DNA/P-Fungi | 383 | 0.000 |
| LINE/Tad1 | 210,000 | 0.027 | DNA/PIF-HarbS | 524 | 0.000 |
| LINE/Zorro | 606 | 0.000 | DNA/PIF-Harbing | 505,059 | 0.064 |
| SINE/5S | 280,784 | 0.036 | DNA/PIF-Harbinger | 6,071,750 | 0.774 |
| SINE/5S-Core-RTE | 1,110 | 0.000 | DNA/PIF-ISL2EU | 261,761 | 0.033 |
| SINE/5S-Deu-L2 | 912,376 | 0.116 | DNA/PiggyBac | 2,993,833 | 0.382 |
| SINE/5S-Sauria-RTE | 34,603 | 0.004 | DNA/Sola | 4,036,044 | 0.514 |
| SINE/7SL | 3,800 | 0.000 | DNA/TcMar | 770,322 | 0.098 |
| SINE/Alu | 125,611 | 0.016 | DNA/TcMar-Ant1 | 1,382 | 0.000 |
| SINE/B2 | 169 | 0.000 | DNA/TcMar-Fot1 | 373,098 | 0.048 |
| SINE/B4 | 14,043 | 0.002 | DNA/TcMar-IS630 | 222 | 0.000 |
| SINE/Core-RTE | 249 | 0.000 | DNA/TcMar-IS885 | 258 | 0.000 |
| SINE/ID | 7,393 | 0.001 | DNA/TcMar-ISRm1 | 140,144 | 0.018 |
| SINE/L2 | 673,312 | 0.086 | DNA/TcMar-ISRm11 | 785,901 | 0.100 |
| SINE/MIR | 65,643,242 | 8.367 | DNA/TcMar-Marin | 129,452 | 0.017 |
| SINE/SINE | 2,023,550 | 0.258 | DNA/TcMar-Mariner | 70,889 | 0.009 |
| SINE/U | 10,544 | 0.001 | DNA/TcMar-Mogwai | 150 | 0.000 |
| SINE/tRNA-7SL | 1,761 | 0.000 | DNA/TcMar-Pogo | 351,739 | 0.045 |
| SINE/tRNA-C | 382,527 | 0.049 | DNA/TcMar-Sagan | 1,253 | 0.000 |
| SINE/tRNA-CR1 | 1,629 | 0.000 | DNA/TcMar-Stowa | 360 | 0.000 |
| SINE/tRNA-Core | 1,582,401 | 0.202 | DNA/TcMar-Stowaway | 306,400 | 0.039 |
| SINE/tRNA-Core-L2 | 682 | 0.000 | DNA/TcMar-Tc1 | 130,082,997 | 16.580 |
| SINE/tRNA-Core-RTE | 418,836 | 0.053 | DNA/TcMar-Tc2 | 1,338,390 | 0.171 |
| SINE/tRNA-Deu | 427 | 0.000 | DNA/TcMar-Tc4 | 394 | 0.000 |
| SINE/tRNA-Deu-CR1 | 263 | 0.000 | DNA/TcMar-Tigge | 77,522 | 0.010 |
| SINE/tRNA-Deu-L2 | 306,972 | 0.039 | DNA/TcMar-Tigger | 960,491 | 0.122 |
| SINE/tRNA-I | 40 | 0.000 | DNA/TcMar-m44 | 718 | 0.000 |
| SINE/tRNA-Jockey | 80 | 0.000 | DNA/Zator | 227,975 | 0.029 |
| SINE/tRNA-L2 | 2,792,247 | 0.356 | DNA/Zisupton | 160,799 | 0.020 |
| SINE/tRNA-Mermaid | 78,248 | 0.010 | DNA/Zisupton-hA | 1,368 | 0.000 |
| SINE/tRNA-RTE | 21,583 | 0.003 | DNA/hAT | 8,420,322 | 1.073 |
| SINE/tRNA-Rex | 376 | 0.000 | DNA/hAT-Ac | 6,490,214 | 0.827 |
| SINE/tRNA-Sauria | 8,075 | 0.001 | DNA/hAT-Blackja | 126,844 | 0.016 |
| SINE/tRNA-Sauria-L2 | 87 | 0.000 | DNA/hAT-Blackjack | 834,760 | 0.106 |
| SINE/tRNA-Sauria-RTE | 67 | 0.000 | DNA/hAT-Charlie | 13,127,185 | 1.673 |
| SINE/tRNA-V | 214,623 | 0.027 | DNA/hAT-Pegasus | 71,608 | 0.009 |
| SINE/tRNA-V-CR1 | 116,925 | 0.015 | DNA/hAT-Restles | 1,242 | 0.000 |
| SINE/tRNA-V-Core-L2 | 2,271 | 0.000 | DNA/hAT-Restless | 750 | 0.000 |
| LTR/Caulimoviru | 1,056 | 0.000 | DNA/hAT-Tag1 | 397,723 | 0.051 |
| LTR/Caulimovirus | 27,087 | 0.003 | DNA/hAT-Tip100 | 8,135,795 | 1.037 |
| LTR/Copia | 1,652,408 | 0.211 | DNA/hAT-Tol2 | 162,206 | 0.021 |
| LTR/DIRS | 26,181,209 | 3.337 | DNA/hAT-hAT1 | 468 | 0.000 |
| LTR/Delta | 834 | 0.000 | DNA/hAT-hAT19 | 1,174,901 | 0.150 |
| LTR/ERV | 342,964 | 0.044 | DNA/hAT-hAT5 | 340,902 | 0.043 |
| LTR/ERV-Foamy | 1,294 | 0.000 | DNA/hAT-hAT6 | 20,682 | 0.003 |
| LTR/ERV-Lenti | 384 | 0.000 | DNA/hAT-hATm | 229,985 | 0.029 |
| LTR/ERV1 | 3,651,606 | 0.465 | DNA/hAT-hATw | 166,440 | 0.021 |
| LTR/ERV4 | 87,149 | 0.011 | DNA/hAT-hATx | 18,402 | 0.002 |
| LTR/ERVK | 1,437,877 | 0.183 | DNA/hAT-hobo | 76,097 | 0.010 |
| LTR/ERVL | 162,909 | 0.021 | Satellite/5S | 74 | 0.000 |
| LTR/ERVL-MaLR | 7,547 | 0.001 | Satellite/Satellite | 5,114,302 | 0.652 |
| LTR/Foamy | 168,391 | 0.021 | Satellite/centr | 10,016 | 0.001 |
| LTR/Gypsy | 74,311,432 | 9.472 | Satellite/telo | 27,225 | 0.003 |
| LTR/Gypsy-Cigr | 4,157,108 | 0.530 | Microsatellite | 16,824,347 | 2.101 |
| LTR/Gypsy-Troyk | 141 | 0.000 | Simple_repeat | 16,487,203 | 2.269 |
| LTR/Gypsy-Troyka | 195 | 0.000 | Unknown/Unknown | 21,135,026 | 2.908 |
| LTR/LTR | 74,290,503 | 9.469 | Other/DNA_virus | 248,853 | 0.034 |
| LTR/Lenti | 2,619 | 0.000 | Other/Other | 119,280 | 0.016 |
| LTR/Ngaro | 4,953,654 | 0.631 | Retroposon/SVA | 40,968 | 0.006 |
| LTR/Pao | 2,064,150 | 0.263 |  |  |  |
| LTR/TATE | 56 | 0.000 |  |  |  |
| LTR/Tate | 192 | 0.000 |  |  |  |
| LTR/Viper | 8,348 | 0.001 |  |  |  |

**Supplementary Table 6.** Summary of gene annotation of the sterlet genome.

| Gene number | 22,184 |
| --- | --- |
| Average gene length | 21,437 bp |
| Average CDS length | 1,553 bp |
| Average exon number | 8.21 |
| Average exon length | 186.30 bp |
| Average intron length | 2,759.48 bp |

**Supplementary Table 7.** Functional annotation of the sterlet genome against different databases.

| **Values** | **Total** | **Swissprot** | **KEGG** | **TrEMBL** | **Interpro** | **Overall** |
| --- | --- | --- | --- | --- | --- | --- |
| Number | 22,184 | 19,507 | 14,754 | 19,231 | 18,521 | 21,089 |
| Percentage | 100% | 87.93% | 66.51% | 86.69% | 83.49% | 95.06% |

**Supplementary Table 8.** Gene family clusters in the sterlet and other vertebrates.

| **Species** | **Gene No.** | **No. of genes in families** | **Unclustered genes** | **Family No.** | **No. of Unique families** | **Average gene No. per family** |
| --- | --- | --- | --- | --- | --- | --- |
| Sterlet | 22,184 | 18,259 | 3,925 | 11,104 | 309 | 1.65 |
| Lamprey | 11,442 | 9,749 | 1,693 | 6,928 | 243 | 1.41 |
| Medaka | 19,699 | 18,327 | 1,372 | 12,950 | 107 | 1.42 |
| Elephant shark | 30,682 | 25,363 | 5,319 | 15,193 | 1,483 | 1.67 |
| Spotted gar | 18,341 | 17,405 | 936 | 13,566 | 51 | 1.28 |
| Stickleback | 20,787 | 19,857 | 930 | 13,885 | 32 | 1.43 |
| Fugu | 18,523 | 18,027 | 496 | 12,653 | 37 | 1.42 |
| Green spotted puffer | 19,583 | 18,004 | 1,579 | 12,730 | 50 | 1.41 |
| Tilapia | 21,437 | 20,788 | 649 | 13,603 | 84 | 1.53 |
| Zebrafish | 26,458 | 24,720 | 1,738 | 14,944 | 184 | 1.65 |
| Atlantic cod | 20,084 | 18,503 | 1,581 | 13,471 | 41 | 1.37 |
| Asian arowana | 22,016 | 18,612 | 3,404 | 12,916 | 21 | 1.44 |
| Coelacanth | 19,568 | 18,247 | 1,321 | 12,855 | 144 | 1.42 |
| Common carp | 75,414 | 59,228 | 16,186 | 29,323 | 4,614 | 2.02 |
| Atlantic Salmon | 109,584 | 99,340 | 10,244 | 32,538 | 7,177 | 3.05 |
